# Likely Autochthonous Transmission of *Trypanosoma cruzi* to Humans, South Central Texas, USA

**DOI:** 10.3201/eid2303.161157

**Published:** 2017-03

**Authors:** Sarah M. Gunter, Kristy O. Murray, Rodion Gorchakov, Rachel Beddard, Susan N. Rossmann, Susan P. Montgomery, Hilda Rivera, Eric L. Brown, David Aguilar, Lawrence E. Widman, Melissa N. Garcia

**Affiliations:** Baylor College of Medicine, Houston, Texas, USA (S.M. Gunter, K.O. Murray, R. Gorchakov, D. Aguilar, M.N. Garcia);; University of Texas School of Public Health, Houston (S.M. Gunter, E.L. Brown);; South Texas Tissue and Blood Center, San Antonio, Texas, USA (R. Beddard);; Gulf Coast Regional Blood Center, Houston (S.N. Rossmann);; Centers for Disease Control and Prevention, Atlanta, Georgia, USA (S.P. Montgomery, H. Rivera);; Cardiac Electrophysiology Consultants of South Texas, PA, San Antonio (L.E. Widman)

**Keywords:** Trypanosoma cruzi, parasites, Chagas disease, autochthonous transmission, humans, blood donors, Texas, United States

## Abstract

Chagas disease, caused by *Trypanosoma cruzi*, is a major neglected tropical disease affecting the Americas. The epidemiology of this disease in the United States is incomplete. We report evidence of likely autochthonous vectorborne transmission of *T. cruzi* and health outcomes in *T. cruzi*–seropositive blood donors in south central Texas, USA.

Chagas disease (*Trypanosoma cruzi* infection) is a neglected tropical disease affecting the Americas and a major cause of preventable illness and death, with ≈6–8 million cases worldwide (*1*). This disease can cause progressive cardiac damage postinfection in 30% of infected persons without any initial suggestive clinical symptoms. These latent infections can remain quiescent for decades before manifesting as cardiac complications, including cardiomyopathy, heart failure, and rare cardiac arrest (*2*).

In 2010, US Food and Drug Administration (Sil-ver Springs, MD, USA) issued final guidelines regard-ing screening of the US blood supply for *T. cruzi* (*3**,**4*). During 2008–2012, screening results showed that 1 in 6,500 donors from an area covering most of the state of Texas were reactive for *T. cruzi* antibodies (*5*). The origin of infection for these donors was unknown. How-ever, high infection rates for reservoir animals and triato-mine bug vectors in south central Texas suggested that *T. cruzi* transmission cycles resulting in human infections could occur at a higher frequency than suspected (*6**,**7*). Therefore, we evaluated potential transmission sources and cardiac health of blood donors from south central Texas with *T. cruzi* antibodies.

## The Study

This study was approved by institutional review boards at Baylor College of Medicine (Houston, TX, USA), the Gulf Coast Regional Blood Center (Houston), and the South Texas Tissue and Blood Center (San Antonio, TX, USA). Blood donors residing in the greater San Antonio, Texas, area who had *T. cruzi* antibodies detected by PRISM Chemiluminescent Immunoassay (Abbott Laboratories, Chicago, IL, USA) or Ortho T. cruzi ELISA (Ortho-Clinical Diagnostics Inc., Raritan, NJ, USA), and a positive result for a Radioimmune Precipitation Assay (Quest Diagnostic Laboratories, Madison, NJ, USA) or an ESA Chagas Test (Abbott Laboratories) during January 1, 2008–December 31, 2014, were invited to participate in the study. Persons previously enrolled in a Houston-based *T. cruzi*–seropositive blood donor project were not eligible for this study (*8*).

Letters in English and Spanish were sent to donors who had *T. cruzi* antibodies by the blood centers for this study. Those who agreed to participate provided informed consent. We performed 3 procedures: 1) blood collection for additional serologic screening, 2) structured interview to assess potential transmission sources and health, and 3) 12-lead resting electrocardiogram (ECG) (*8*).

Blood specimens were used for serologic testing (Table 1). We defined a case of *T. cruzi* infection if donor screening test results and >2 serologic test results were positive. Likely autochthonous *T. cruzi* infection was defined in a case-patient who had no major travel to a Latin American country (lasting >2 weeks or that included an overnight stay in a rural region), not having been born in Latin America, and not having a mother born in Latin America (*4**,**8**–**10*). Congenital transmission from a maternal grandmother (2 contiguous congenital infections) cannot be ruled out with this case definition but is unlikely given the low risk for congenital transmission (*7*). Occupations, residential history, and clinical health information were reviewed in a questionnaire. ECG readings were interpreted by a board-certified cardiologist.

**Table 1:** Characteristics for 14 blood donors infected with *Trypanosoma cruzi*, south central Texas*

Donor no./age, y/sex	Likely autochthonous transmission†	Blood bank serologic test results‡			Study serologic test results§					ECG results¶	Concurent condition
		PRISM or ORTHO	RIPA or ESA		Hemagen	Stat-Pak	DPP	EIA	TESA		
1/83/M	Yes	+	+		+	+	+	+	+	Primary AV block, atypical incomplete right BBB, lateral asymmetric T inversion	None
2/61/F	Yes	+	+		+	+	+	+	+	Inferolateral asymmetric T inversion	Hypertension
3/71/M	Yes	+	+		+	+	+	+	+	LAD, nonspecific ST/T wave abnormality	Kidney failure, hypertension
5/19/M	Yes	+	+		+	+	+	+	+	Normal	None
6/60/M	Yes	+	+		+	+	+	+	+	Primary AV block	Diabetes, hypertension
7/56/F	Yes	+	+		+	+	+	–	+	Minimum voltage criteria for LVH	None
8/52/M	Yes	+	+		+	+	+	+	+	LAD	Parkinson’s disease
9/25/F	Yes	+	+		+	+	+	+	+	Normal	None
10/51/F	Yes	+	+		+	+	+	Ind	+	Normal	Heart attack
11/52/F	Yes	+	+		+	+	+	+	+	Normal	None
12/45/M	No	+	+		+	+	+	+	+	Normal	Borderline diabetes
13/35/F	No	+	+		+	+	+	+	+	Normal	None
14/34/F	No	+	+		Ind	+	+	+	+	Nonspecific T wave change	None

*Demographic information, likely autochthonous transmission, and concurrent conditions were determined through case-patient interview. ECG, electrocardiogam; Ind, indeterminate; +, positive; –, negative. Test results were based on manufacturers’ protocols for serologic testing.  †Donors listed as showing autochthonous transmission (donors 12–14) reported living in Mexico or Chile.  ‡ESA, Chagas Test (Abbott Laboratories, Chicago, IL, USA); ORTHO, *T. cruzi* ELISA (Ortho-Clinical Diagnostics Inc., Raritan, NJ, USA); PRISM, Chemiluminescent Immunoassay (Abbott Laboratories); RIPA, radioimmune precipitation assay (Quest Diagnostic Laboratories, Madison, NJ, USA). §DPP, dual path platform immunochromatographic confirmation assay (Chembio, Medford, NY, USA); EIA, Chagatest recombinant v3.0 enzyme inmmunoassay (Wiener, Rosario, Argentina); Hemagen; Chagas EIA Kit (Hemagen Diagnostics, Inc., Columbia, MD, USA); Stat-Pak, Chagas immunochromatographic sssay (Chembio, Medford, NY, USA); TESA, trypomastigote excreted or secreted antigen immunoblot (bioMérieux, Marcy l’Etoile, France). Hemagen, Stat-Pak, and DPP were performed at Baylor College of Medicine, (Houston, TX, USA), and EIA and TESA were performed at the Centers for Disease Control and Prevention (Atlanta, GA, USA).  ¶Results were determined from readout of a resting 12-lead ECG and interpreted by a board-certified cardiologist. AV, atrioventricular; LAD, left axis deviation; LVH, left ventricular hypertrophy; RBBB, right bundle branch block.

For persons who donated blood in the greater San Antonio area during the study period, we found that 61/256,801 donors had positive serologic results for *T. cruzi* infection (1/4,200 donors had positive serologic results for *T. cruzi* infection by 2 assays). Seventeen (28%) of these were enrolled in the study; additional serologic testing confirmed that 14 had antibodies against *T. cruzi* when the study began (Table 1). These persons had a mean age of 47 years (range 19–83 years); 50% were Hispanic, 50% were non-Hispanic white, and 50% were men. For 3 persons whose blood donor testing results were not confirmed by further serologic testing, 2 were non-Hispanic and 1 was Hispanic (2 women and 1 man); mean age was 51 years. Because of the blinded nature of study recruitment, we cannot identify demographic data for persons who received the letter and chose not to participate.

Likely autochthonous transmission of *T. cruzi* was suspected for 11 (79%) of 14 persons, as defined by study criteria. These 11 persons had a mean age of 50 years; 7 were non-Hispanic whites, and 6 were men. Remaining data presented will concern only the 11 newly identified persons with likely autochthonous infections.

A structured interview adapted from a questionnaire used by the Centers for Disease Control and Prevention (Atlanta, GA, USA), the American Red Cross (Washington, DC, USA), and Blood Systems, Inc. (Scottsdale, AZ, USA), was used to identify risk factors for *T. cruzi* infection (*4*). Because of the lifelong nature of infection and antibody-based diagnostics used, a specific time of infection could not be established for each case-patient. However, we identified common themes for transmission risks for this cohort. Most (91%) case-patients with likely autochthonous infection reported a history of living in a rural community (Figure). Residence in rural communities could pose a risk for *T. cruzi* transmission because this setting might lead to close proximity with sylvatic transmission cycles involving the vector and infected animals (*11*).

**Figure:**
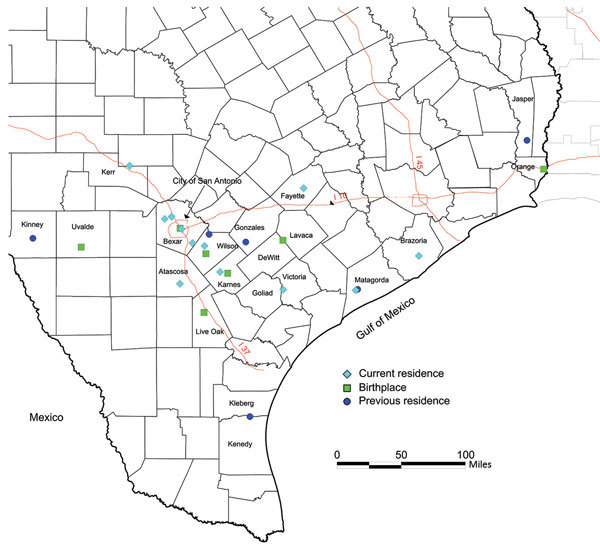
Current and previous residences of persons with likely autochthonous infection with *Trypanosoma cruzi*, south central Texas, USA, including 11 autochthonous donors with current residence and birthplace. County boundaries are shown. Previous residences in Texas were chosen if the case-patient reported living in the location >5 years.

Although recreational activities or occupations associated with outdoor exposure were reported among our cohort, we obtained evidence suggesting that opportunities for transmission might be occurring near homes in rural communities (Table 2). Specifically, patients with likely autochthonous infections reported seeing the vector around their current or previous residence (36%), and had animal housing near their homes (73%). An extensive history of outdoor recreational activities of hunting and camping, which has been suggested as a high-risk activity for *T. cruzi* transmission in the southern United States, was less common then expected (36%) (*8**,**12*). Two of 11 case-patients reported agricultural jobs and staying in substandard housing during the harvest season, thereby introducing the potential for disease transmission from triatomines in the home.

**Table 2:** High-risk activity profile for 11 case-patients with likely autochthonous infection with *Trypanosoma cruzi*, south central Texas, USA*

Case-patient	Birthplace/former residence	Current residence	Occupational	Recreational camping	Recreational hunting
1	+++	+	++	++	+
2	+++	0	0	+	0
3	+	+	+++	+	+
4	0	0	0	++	0
5	+++	0	+	+	0
6	+++	+++	+	0	++
7	+	+++	+++	+	0
8	++	0	++	0	++
9	0	+++	+	0	0
10	+	+	0	0	0
11	++	+++	0	+	0

*Risk was determined through administration of a patient survey. No risk (0) was defined as not living in a rural area and having no history of outdoor occupation or recreational activities. Low risk (+) was defined as ever living in a rural area, having an outdoor occupation, or engaging in hunting or camping in an area with known triatomine activity. Moderate risk (++) was defined as, in addition to low-risk activities, an extensive history of these activities (>1 y), or having slept in a tent in a rural part of Texas. High risk (+++) was defined as, in addition to moderate-risk activities, reporting 1 of the following: reported seeing triatomines, had collective animal housing around the property, or lived or slept in substandard housing.

Five case-patients reported a lack of knowledge of Chagas disease by their primary care physicians. Some case-patients were provided with misinformation, reporting having been told that their screening test result must be false positive because they had no travel history. Furthermore, only 2 case-patients were offered treatment before enrollment in the study. One case-patient reported that, despite seeking treatment for >1 year, he was unable to find a physician able and willing to help.

This finding is particularly problematic given that a large proportion (6 of 11) of this cohort had abnormal ECG readings possibly attributable to Chagasic cardiac disease. Although precise cardiac etiologies could not be determined, prevalence of ECG abnormalities was higher than that for population-based studies (*13**,**14*); common findings included atrioventricular block and left axis deviation (Table 1). A previous report also highlighted the same lack of physician awareness of Chagas disease in Texas, despite patients having positive serologic screening results and cardiac manifestations (*8*).

## Conclusions

Given the low level of participation of seropositive blood donors, results of this study are limited to persons who participated and might not represent the larger Texas blood donor population or general population. Also, because a 7-year span separated initial screening and enrollment in this study, it is difficult to identify why 3 persons who were initially positive by blood bank screening had discordant results during the study. At follow-up, participating persons were tested with available Centers for Disease Control and Prevention assays, Food and Drug Administration–approved screening, or supplemental tests.

Our study adds 11 cases of likely domestically acquired *T. cruzi* infection to the increasing body of evidence for autochthonous Chagas disease transmission in the southern United States. Combined with previous studies indicating a high rate of *T. cruzi* infection in triatomine vectors and mammalian reservoirs in this area, our study shows that south central Texas could be a focal point for endemic disease transmission (*7*,*15*). We also identified a major knowledge gap for Chagas disease, which highlights the need for enhanced public health campaigns targeting clinicians and the general population in south central Texas.
